# Comparison of adjuvant emulsions for their safety and ability to enhance the antibody response in horses immunized with African snake venoms

**DOI:** 10.1016/j.jvacx.2022.100233

**Published:** 2022-10-25

**Authors:** Mauricio Arguedas, Deibid Umaña, Edwin Moscoso, Armando García, Carolina Pereira, Andrés Sánchez, Gina Durán, Daniel Cordero, Adriana Sánchez, Álvaro Segura, Mariángela Vargas, María Herrera, Mauren Villalta, Aarón Gómez, Catalina Salas, Cecilia Díaz, José María Gutiérrez, Guillermo León

**Affiliations:** a Instituto Clodomiro Picado, Facultad de Microbiología, Universidad de Costa Rica, San José, Costa Rica; b Escuela de Zootecnia, Facultad de Ciencias Agroalimentarias, Universidad de Costa Rica, San José, Costa Rica

**Keywords:** Adjuvant emulsions, Antivenom, Carbigen, Emulsigen-D, Freund adjuvants, Horse immunization, Montanide, Snake venom

## Abstract

Adjuvant emulsions are widely used to enhance the antibody response in animals used as immunoglobulin source to produce snake antivenoms. We tested the performance of four commercial emulsion adjuvants (Montanide, Freund, Carbigen, and Emulsigen-D) and an experimental adjuvant (QH-769) in the antibody response of horses towards venoms of the African snakes *Bitis arietans*, *Echis ocellatus*, *Dendroaspis polylepis* and *Naja nigricollis*. Montanide, Freund and Carbigen adjuvants generated the highest immune response but induced moderate/severe local lesions at the site of injection. In contrast, Emulsigen-D and QH-769 adjuvants generated the lowest immune response and low incidence of local lesions. No evidence of systemic alterations was observed in the horses immunized with any of the adjuvants. It is suggested that the use of Montanide or Freund-based emulsions in the first immunization steps, followed by the use of Emulsigen-D, QH-769 or similar adjuvants in the following injections, could result in a satisfactory immune response against snake venoms, while not inducing serious local deleterious effects.

## 1 Introduction

Snake antivenoms are formulations of immunoglobulins, or immunoglobulin fragments, purified from the plasma of animals (mainly horses or sheep) immunized with snake venoms [1]. Owing to the ability of these immunoglobulins to bind venom toxins and neutralize their toxicity, antivenoms have been used for over a century for the medical treatment of snakebite envenomations [1].

Immunization of animals for antivenom production is carried out by the repeated injection of various doses of venoms from one or more venomous species, following a predefined immunization program. The amount of venom injected has to be low in order to prevent tissue damage or other manifestations of envenomation, but high enough to trigger the activation of the immune system to produce large quantities of anti-venom antibodies [2], [3]. In horses, injections are normally applied by the subcutaneous (s.c.) route and generally contain between 0.5 and 5.0 mg of venom per injection, depending on the manufacturer [3].

The goal of immunization schemes is to generate a high level of neutralizing antibodies, so that antivenoms can be formulated at a high potency and low total protein content, thus rendering the products more effective and safer. Moreover, with high anti-venom antibody titers more vials of antivenom can be produced per plasma volume, reducing the relative weight of fixed costs, thus making the process more productive and efficient [4].

Generally, during immunization, venoms are mixed with adjuvant substances that enhance the antibody response of animals. Several studies have evaluated different adjuvants and immunization schemes for antivenom production [5], [6], [7], [8], [9], [10], but more research is needed in this field. The adjuvants normally used in the production of antivenoms are mineral salts, polymers, or emulsions [2], [3].

Emulsions are mixtures composed of two or more immiscible liquids whose interfaces are stabilized by surfactants to form heterogeneous systems of microdroplets of a discontinuous phase dispersed in a continuous dispersing phase [11]. Depending on how the phases are organized, emulsions can be: a) simple water-in-oil (w/o) emulsions, when an aqueous phase is dispersed in an oily phase; b) simple o/w emulsions, when an oily phase is dispersed in an aqueous phase; c) multiple w/o/w emulsions, when a simple w/o emulsion is dispersed in an aqueous phase; or d) multiple o/w/o emulsions, when a simple o/w emulsion is dispersed in an oily phase [11].

The adjuvant activity of emulsions operates by a complex mechanism that is not entirely understood. It has been suggested that it involves the ability of emulsions to form depots from where immunogens are slowly released, generating a sustained stimulus to the immune system [12]. Other researchers indicate that adjuvant activity of emulsions is due to their ability to induce apoptosis or necrosis in cells which respond by generating “danger” signals that trigger the immune response [13]. Thus, the explanation of how adjuvant emulsions operate remains controversial.

Several adjuvant emulsions are commercially available. Montanide ISA 50 V2 is composed of an injectable mineral oil, vegetable oleic acid and a mannitol derived emulsifier. Incomplete Freund adjuvant is composed of mineral oil and Arlacel A as emulsifier agent. Complete Freund adjuvant contains, in addition, heat-killed cells of *Mycobacterium tuberculosis* or *M. butyricum*. Carbigen is an emulsified suspension of Carbopol 934P. Emulsigen-D is an o/w adjuvant that contains dimethyl octadecyl ammonium bromide (DDA) incorporated into the emulsion. Technical sheets of these commercial products can be found in the web. In addition, QH-769 is a squalene-based formulation used to prepare w/o emulsions.

Previously, we used a mouse model to study the relationship between the physical characteristics of emulsions and their adjuvant activities on the antibody response towards the venom of the African viperid snake *Echis ocellatus* [5]. Now, we scaled up the model to horses and tested the performance of four commercial emulsion adjuvants (i.e., Montanide, Freund, Carbigen, and Emulsigen-D) and an experimental adjuvant (i.e., QH-769) in the antibody response towards venoms of the African snakes *E. ocellatus, Bitis arietans*, *Dendroaspis polylepis* and *Naja nigricollis*.

## 2 Materials and methods

All procedures carried out in this study meet the International Guiding Principles for Biomedical Research Involving Animals [14]. All procedures involving animals were approved by the Institutional Committee for the Care and Use of Laboratory Animals of Universidad de Costa Rica (approval code CICUA 202-2020).

### 2.1 Snake venoms

Venoms of adult specimens of *B. arietans* (batch #322.061), *E. ocellatus* (batch #200.171), *D. polylepis* (batch #416.031) and *N. nigricollis* (batch #616.031) were purchased from Latoxan (Portes-dès Valence, France). After collection, venoms were stabilized by lyophilization and stored at −40 °C. Solutions of venoms were prepared immediately before use.

### 2.2 Immunologic adjuvants

The adjuvants used in this study were: Montanide ISA 50V2 (SEPPIC, Castres, France, batch 190301019150); Complete Freund (Sigma-Aldrich, St. Louis. MO, USA, batch SLCJ8308); Carbigen (MVP Adjuvants, Teaneck, NJ, USA; batch F18123); Emulsigen-D (MVP Adjuvants, batch D01449) and QH-769 (IDRI, Seattle, WA, USA, experimental batch). All adjuvants were used according to the manufacturers’ instructions.

### 2.3 Immunization of horses

Five groups of four Spanish horses (370–490 kg body weight) were immunized using periodic injections of immunogens composed of equal parts of venoms of *B. arietans*, *E. ocellatus*, *D. polylepis* and *N. nigricollis*, dissolved in 0.12 M NaCl, 0.04 M phosphates, pH 7.2 (PBS) and mixed with either Montanide, Freund, Carbigen, Emulsigen-D or QH-769 adjuvants. In each group of horses, the same adjuvant was used throughout the immunization in all injections. The immunogens were administered in a single injection site in the back of the horse by the s.c. route. Doses and schedule of injections are specified in Table 1. The total volume of each venom injection was 2 mL. Blood samples were collected from the jugular vein before the onset of immunization and at different times during immunization to determine hematological, plasma chemistry and immunological parameters. The clinical and physical status of the animals were constantly monitored throughout the procedure. The whole process was carried out under veterinary supervision.

**Table 1:** Immunization scheme* and bleeding program.

Day	Venom (mg)	Injection volume (mL)
1	0.5	2.0
15	0.5	2.0
23	1.0	2.0
43	1.0	2.0
60	2.0	2.0
71	3.0	2.0
78	Bleeding	

* In each group of horses, the same adjuvant was used throughout the immunization in all injections.

### 2.4 Body condition, hematological and plasma chemistry evaluation

Body condition score (BCS) of horses was determined by palpation and visual appraisal of the fat accumulation in anatomical areas such as behind the shoulders, over the ribs, along the neck, along the withers, the crease down back and the tailhead [15]. Scores range from 1 (extremely emaciated) to 9 (extremely fat), with an ideal range between 4 and 6. Lesions developed at the injection site were evaluated and classified according to their severity as explained in the Results and Discussion section. Hematological analyses (i.e., erythrocyte, leukocyte and platelet counts, and hemoglobin concentration) were carried out in a Veterinary Hematology Analyzer (Exigo Eos Hematology System; Boule Diagnostics AB, Stockholm, Sweden). Plasma chemistry analyses were carried out in a clinical chemistry analyzer (Spin200E Automatic biochemistry analyzer; Spinreact, Barcelona, España). Creatine kinase (CK) was determined by the corresponding International Federation of Clinical Chemistry and Laboratory Medicine (IFCC) method. Creatinine was determined by a kinetic modification of the Jaffe colorimetric method [16] and urea by a modification of the Talke and Schubert method [17]. Aspartate transaminase (AST) and alkaline phosphatase (ALP) were determined by the corresponding IFCC methods. Gamma-glutamyl transferase (GGT) was determined by a modification of the Szasz procedure [18]. Total protein concentration was determined by the Biuret method [19], and albumin concentration by the bromocresol green colorimetric method [20].

### 2.5 Venom fractionation by FPLC

Fifteen milligrams of venoms of *D. polylepis* and *N. nigricollis* were fractionated by size exclusion chromatography in an ÄKTA FPLC system equipped with a Superdex 200 column. The flow rate was set to 1 mL/min and a mobile phase composed of 5 mM Tris-HCl, 150 mM NaCl, pH 7.5 was used for separation. Protein peaks detected at 280 nm were collected and stored at −20 °C until analysis.

### 2.6 Reactivity of equine sera by enzyme-linked immunosorbent assay (ELISA)

Determination of serum concentration of anti-venom antibodies was done in serum samples from each horse at different points during the immunization scheme. In some experiments, polystyrene plates were coated overnight at room temperature with 100 µL of PBS containing 3 μg of venom of either *B. arietans*, *E. ocellatus*, *D. polylepis* or *N. nigricollis*. In other experiments, plates were coated with 100 µL of PBS containing 32 µg or 7.5 µg of low molecular mass FPLC fractions (<30 kDa) of venoms of *D. polylepis* or *N. nigricollis*, respectively. After washing the plates five times with distilled water, 100 µL of 1:1000 dilution of sera from immunized horses, in PBS-2 % bovine serum albumin (BSA), were added. The plates were incubated for 1 h at room temperature and washed five times. Afterwards, 100 µL of goat anti-horse IgG conjugated with peroxidase, diluted 1:5000 with PBS-2 % BSA, were added to each well. Again, microplates were incubated for 1 h at room temperature. After a final washing step, color was developed by the addition of H_2_O_2_ and *o*-phenylenediamine. Color development was stopped by the addition of 1.0 M HCl. Absorbances at 492 nm were recorded. The relative concentration of anti-venom antibodies (percentage) in serum samples was calculated by interpolation of their absorbances in a calibration curve constructed by using a reference pool of hyperimmune equine plasma used for the commercial production of the antivenom EchiTab-plus-ICP. This hyperimmune plasma is generated by the immunization of horses with the four venoms used in this study. Results were expressed as average ± SD of all horses in each group.

### 2.7 Reactivity of equine sera by Western blot

Venoms of *B. arietans*, *E. ocellatus*, *D. polylepis* and *N. nigricollis* were separated in SDS-PAGE, under non-reducing conditions, using a 12 % acrylamide concentration [21]. Gels were transferred to a nitrocellulose membrane at 30 mAmp overnight. Then, the membranes were blocked with PBS-1 % casein for 30 min. Next, membranes were incubated for 1 h with a pool of serum samples of all horses in each group, diluted 1/1000 with PBS-0.1 % casein. After washing the membranes three times with PBS-2 % casein, they were incubated for 1 h with goat anti-horse IgG conjugated with peroxidase, diluted 1:2000 with PBS-0.1 % BSA. Finally, after the last washing step, 4-chloro-1-naphthol color development substrate was added, and the reaction was stopped with distilled water. Families of the recognized bands were identified by comparing the electropherograms with those previously published for *B. a. arietans* [22], *E. ocellatus* [23], *D. polylepis* [24] and *N. nigricollis* [25].

### 2.8 Neutralization of lethal activity

Groups of five CD-1 mice were pretreated with tramadol s.c., at a dose of 50 mg/kg, to reduce pain during the test [26]. Fifteen minutes later, mice received an intravenous (i.v.) injection of 0.2 mL of solutions containing different mixtures of a challenge dose of venom with variable dilutions of a pool of antisera of horses from each group. Challenge doses corresponded to 5 LD_50_s for the venoms of *B. arietans* and *E. ocellatus*, and 2 LD_50_s for the venoms of *D. polylepis* and *N. nigricollis*. Saline solution was used as solvent for antisera dilutions. The mixtures were incubated at 37 °C for 30 min before injection. The number of deaths during the following 6 h were recorded [27] and used to estimate the median effective dose (ED_50_, i.e., the ratio mg venom/mL antiserum at which 50 % of the challenged mice survived) by Probits [28]. Surviving mice were euthanized by CO_2_ inhalation. Results were reported as ED_50_, expressed as mg venom per mL antiserum, and the corresponding 95 % confidence interval (95 % CI).

### 2.9 Statistical analyses

The plasma chemistry values were assessed by one-way ANOVA. For values of hematology parameters and antibody concentration, a general linear model of repeated measures test was used, and when the sphericity was not met, the Greenhouse-Geisser correction factor was used. A p-value < 0.05 was considered significant. In the case of the neutralization of the lethal activity, groups having non-overlapping values of the 95 % confidence intervals were considered significantly different.

## 3 Results and discussion

### 3.1 Clinical and laboratory alterations

Before immunization, all horses had an adequate health status, i.e., 370–490 kg body weight, horse body condition score between 5.5 and 6.5, normal hematology (Supplementary Table S1) and plasma chemistry parameters (Table 2), and no apparent clinical problems. Fourteen days after each immunogen injection, we observed the local lesions that developed at the site of immunogen injection.

**Table 2:** Plasma chemistry analyses of horses immunized with venoms using different adjuvants.

Adjuvant	Day	CK^1^	Creatinine^2^	Urea^3^	AST^4^	ALP^5^	GGT^6^	Total protein^7^	Albumin^8^
Montanide	0	251 ± 88	120 ± 10	8 ± 1	366 ± 72	174 ± 41	23 ± 8	72 ± 3	32 ± 2
	84	93 ± 26	81 ± 5*	6 ± 1*	217 ± 51*	209 ± 87	23 ± 12	91 ± 9*	23 ± 3*
Freund	0	264 ± 48	129 ± 24	9 ± 2	386 ± 23	208 ± 32	17 ± 2	72 ± 6	32 ± 1
	84	98 ± 17	90 ± 26*	7 ± 2*	225 ± 20*	224 ± 27	21 ± 8	88 ± 3*	25 ± 1*
Carbigen	0	257 ± 107	103 ± 10	7 ± 1	361 ± 52	261 ± 19	28 ± 15	72 ± 3	32 ± 2
	84	229 ± 114	92 ± 18*	7 ± 1	261 ± 46*	270 ± 84	17 ± 3	80 ± 5	27 ± 0
Emulsigen D	0	257 ± 9	92 ± 55	8 ± 1	376 ± 58	208 ± 14	18 ± 2	72 ± 2	32 ± 2
	84	162 ± 16	90 ± 9*	7 ± 0*	314 ± 138*	220 ± 82	14 ± 3	84 ± 1*	27 ± 1*
QH769	0	328 ± 75	111 ± 12	8 ± 1	356 ± 44	208 ± 34	22 ± 7	72 ± 7	31 ± 1
	84	392 ± 269	100 ± 18*	8 ± 1	313 ± 57*	225 ± 69	18 ± 4	77 ± 1	29 ± 2

1 CK: Creatine kinase. Values are expressed as IU/L, and correspond to the average ± SD.

2 Creatinine. Values are expressed as μmol/L, and correspond to the average ± SD.

3 Urea. Values are expressed as mmol/L, and correspond to the average ± SD.

4 AST: Aspartate transaminase. Values are expressed as IU/L, and correspond to the average ± SD.

5 ALP: Alkaline phosphatase. Values are expressed as IU/L, and correspond to the average ± SD.

6 GGT: Gamma-glutamyl transferase. Values are expressed as IU/L, and correspond to the average ± SD.

7 Total protein. Values are expressed as g/L, and correspond to the average ± SD.

8 Albumin. Values are expressed as g/L, and correspond to the average ± SD.

* Significantly different (p < 0.05) when comparing samples collected at day 84 (at the end of immunization) with samples collected at time 0 (before immunization).

**Fig. 1:**
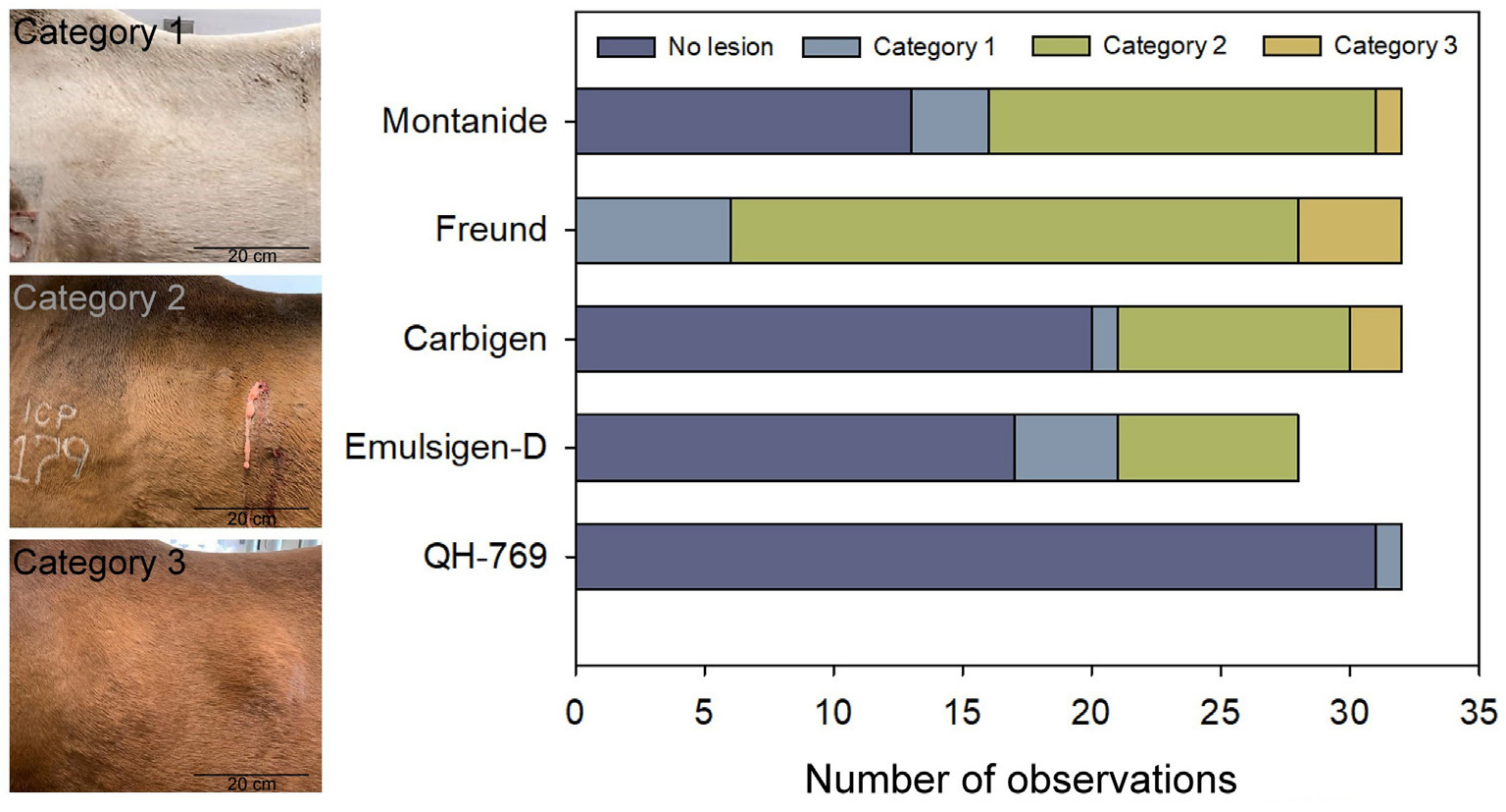
Record of local lesions induced in horses by the injection of a mixture of venoms of *B. arietans*, *E. ocellatus*, *D. polylepis*, and *N. nigricollis* emulsified in different adjuvants. Each observation corresponds to an individual horse 14 days after every injection of venom with adjuvants (see schedule in Table 1). Category 1 includes mild painful lesions, characterized by a diffuse edema around the injection site, which are totally reabsorbed in the following 3–4 weeks. Category 2 includes well-circumscribed lesions associated with mild/moderate pain and are characterized by the formation of soft consistency abscesses with thinned skin spots which eventually ulcerates and/or opens fistula through which a bloody pus-like material is discharged. These lesions develop scar tissue and heal in 4–8 weeks. Category 3 comprises well circumscribed or diffused lesions that induce moderate/severe pain and are characterized by the development of fibrous tissue (solid to palpation). These lesions may or may not involve fistula formation before fibrosis and are usually healed and reabsorbed in 4–10 weeks. The photographs correspond to injections applied on the anterior/left part of the back, with the head of the animals towards the left of the image.

Local lesions varied in size (from 0 to 20 cm diameter). We classified these lesions in three categories, depending on their pathological status (Fig. 1): Category 1 includes mild painful lesions (as judged by the reaction to palpation), characterized by a diffuse edema around the injection site, which are totally reabsorbed in the following 3–4 weeks. Category 2 includes well-circumscribed lesions associated with mild/moderate pain and characterized by the formation of soft consistency abscesses with thinned skin spots which eventually ulcerates and/or develops fistula through which a bloody pus-like material is discharged. These lesions develop scar tissue and heal in 4–8 weeks. Category 3 comprises well circumscribed or diffused lesions associated with moderate/severe pain and characterized by the development of fibrous tissue (solid to palpation). These lesions may or may not involve fistula formation before fibrosis and are usually healed and reabsorbed in 4–10 weeks.

More abundant and severe local lesions developed in the horses immunized with Montanide, Freund, and Carbigen adjuvants. These lesions were less frequent and severe in horses immunized with Emulsigen-D and almost absent in the horses immunized with QH-769 (Fig. 1). After the fourth booster, horse #171 had to be withdrawn from the study due to health problems not related to the immunization procedures. Therefore, the Emulsigen-D group has four missing observations (Fig. 1).

Tissue damage in the site of venom immunogen injection is likely to be due to a combination of a) the irritation of the surrounding tissue produced by the adjuvant, b) the toxic effects of the venom that is slowly released from the emulsion, and c) the activities triggered by inflammatory cells stimulated by venom and adjuvants [3]. However, our results suggest that the major contribution to the formation of local lesions is due to the adjuvants since the amount of venom was the same in all cases despite evident differences in the extent of local tissue damage depending on the adjuvant used.

Venoms of *Echis spp* have demonstrated their potential to induce local muscle damage during immunization in a rabbit model [29]. However, no significant increments in the plasma concentration of CK (F = 3.020 _(1; 36)_, p = 0.091) were observed in these horse groups (Table 2), which indicates that the mass of venoms injected to horses in this study is not high enough to induce significant muscle damage. This result contrasts with a previous report in which higher doses (1.5–50.0 mg) of a mixture of venoms of *Bothrops asper*, *Crotalus simus*, *Lachesis stenophrys*, mixed with Freund or sodium alginate adjuvants, resulted in a small but significant rise of the plasma concentration of CK [30].

Systemic signs of envenomation (i.e., bleeding, hypotension, fever, hematuria, or neurotoxicity) were not observed in any of the groups. Moreover, no significant alterations of hematocrit (F = 1.608 _(16.38; 57.33)_, p = 0.095) or hemoglobin (F = 1.568 _(17.46; 61.12)_, p = 0.100) values, or erythrocyte (F = 1.759 _(15.81; 55.35)_, p = 0.063), leukocyte (F = 1.064 _(16.11; 56.41)_, p = 0.410) or platelet (F = 1.311 _(13.89; 48.61)_, p = 0.236) counts were observed during immunization (Supplementary Table S1). Although significant differences in values of creatinine (F = 7.591 _(1; 37)_, p = 0.009) and a slight variation in urea concentration (F = 4.230 _(1; 37)_, p = 0.047) were observed (Table 2), the concentrations of these analytes were within the normal ranges of the local horse population (i.e., 61–150 μmol/L and 4–8 mmol/L, respectively), indicating that no kidney injury occurred during immunization. Moreover, when significant differences were observed, values at the end of immunization were generally lower than those before the immunization, hence indicating that no increments in these analytes occurred.

In contrast to our previous results obtained in a rabbit model [29], no evident hepatic damage was induced during the immunization. Despite the fact that AST plasma concentration showed a significant difference at the end of the immunization scheme (F = 29.080 _(1; 37)_, p < 0.0001) (Table 2), the upper limit of the normal interval for this enzyme (i.e., 449 IU/L) was not exceeded. Moreover, no significant changes in plasma concentration of ALP (F = 1.088 _(1; 37)_, p = 0.304) and GGT (F = 1.026 _(1; 36)_, p = 0.318) were observed during immunization (Table 2).

Overall, our observations indicate that, with the venom doses used in the immunization scheme, no systemic effects of envenomation developed in the immunized horses. The main impact of immunization in the horses’ health was related to the local tissue alterations, mostly due to the action of some adjuvants.

A significant increment in the total protein concentration in plasma was observed in horses immunized with Montanide (F = 16.194 _(1; 6)_, p = 0.007), Freund (F = 23.088 _(1; 6)_, p = 0.003) and Emulsigen-D (F = 58.435 _(1; 6)_, p = 0.001) adjuvants. This finding is explained by a rise in the plasma concentration of globulins (F = 59.051 _(1; 37)_, p < 0.0001) (particularly anti-venom immunoglobulins), which is partially compensated by a decrease of the plasma albumin concentration (F = 59.797 _(1;37)_, p < 0.0001). A similar change in the globulin/albumin ratio has been described in previous studies in horses immunized with viperid snake venoms from Costa Rica [31].

### 3.2 Antibody response

**Fig. 2:**
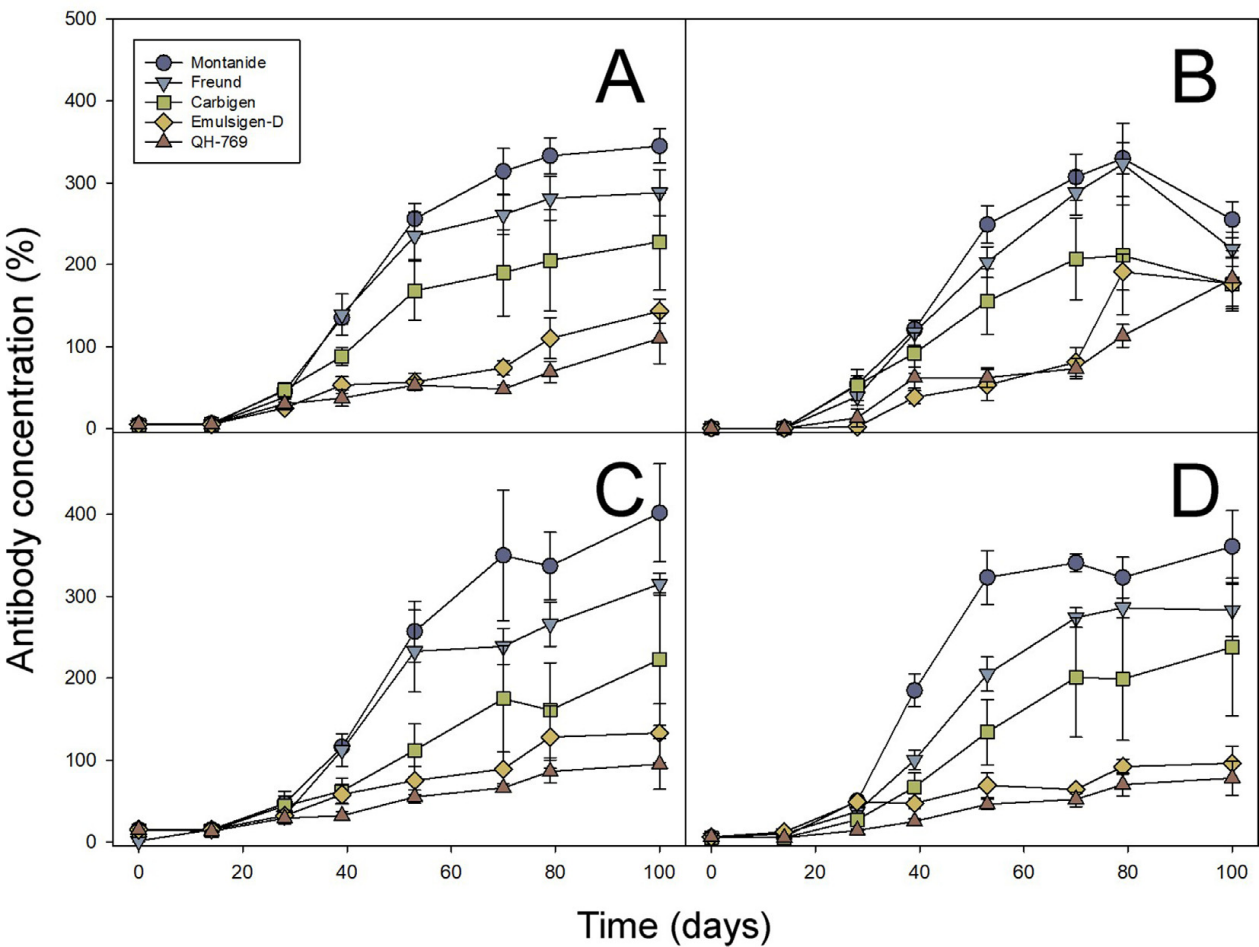
ELISA determination of the antibody response of horses throughout the immunization process. A) anti-*B. arietans* antibodies, B) anti-*E. ocellatus* antibodies, C) anti-*D. polylepis* antibodies, and D) anti-*N. nigricollis* antibodies. ELISA results are expressed as percentage, considering as 100 % the titer of a reference pool of hyperimmune equine sera used for the commercial production of the antivenom EchiTab-plus-ICP. Values correspond to the mean ± standard error (n = 4).

Immunization of horses resulted in a gradual increase of the serum concentration of anti-venom immunoglobulins, as judged by ELISA. Montanide (F = 202.533 (_1.7; 20.7_), p < 0.0001) and Freund (F = 189.904 (_3.4; 40.8_), p < 0.0001) were the adjuvants with the highest ability to enhance the plasma concentration of anti-venom antibodies towards the four venoms. Carbigen (F = 33.537 (_1.2; 15.2_), p < 0.0001) showed an intermediate performance, while Emulsigen-D (F = 66.597 (_2.1; 17.2_), p < 0.0001) and QH-679 (F = 40.787 (_1.3; 16.0_), p < 0.0001) induced the lowest antibody titers (Fig. 2).

**Fig. 3:**
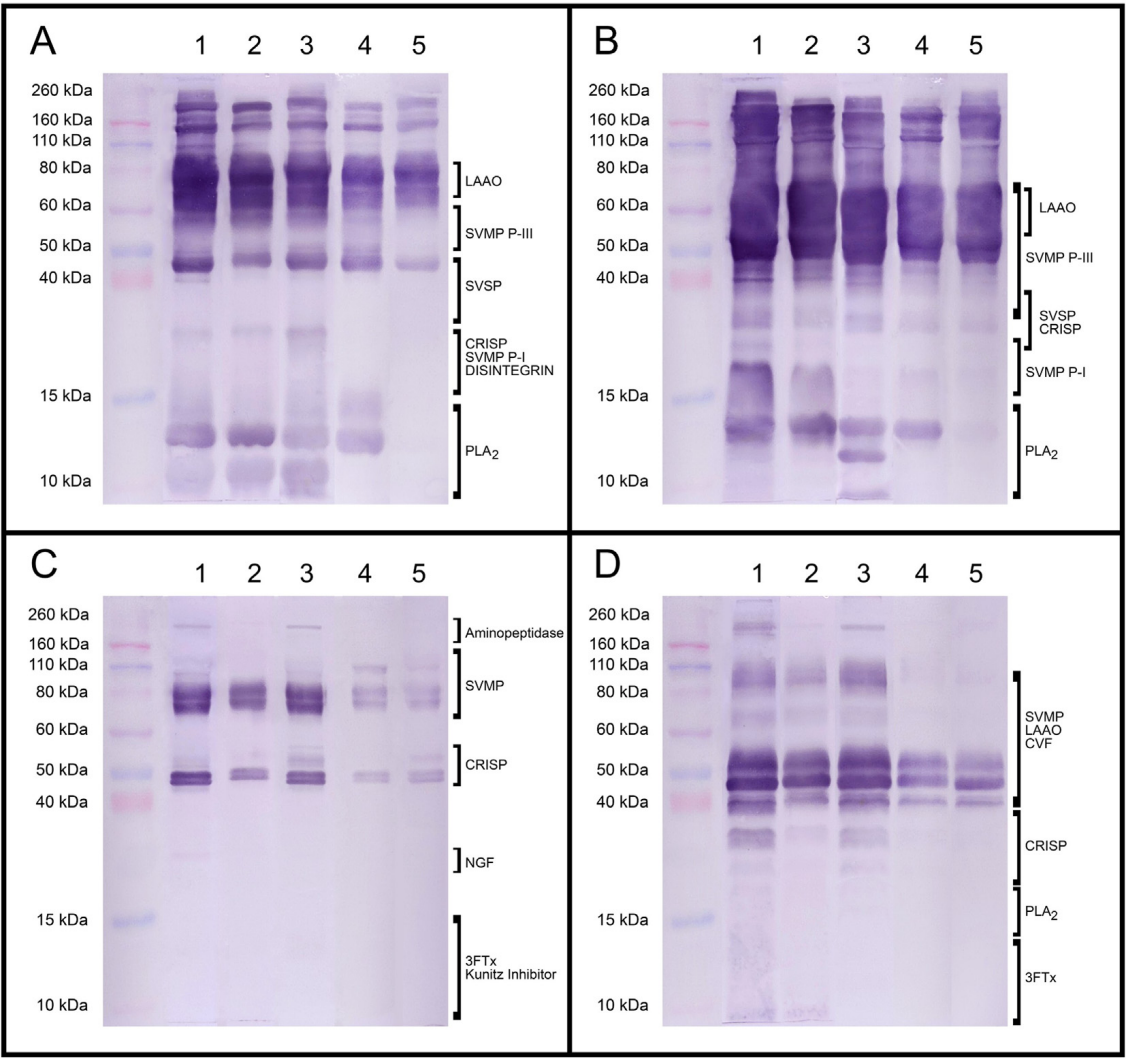
Western blot analysis of the immunoreactivity profile against individual venoms of sera of horses immunized with a mixture of venoms of *B. arietans*, *E. ocellatus*, *D. polylepis*, and *N. nigricollis* emulsified in different adjuvants. Venoms correspond to A) *B. arietans*, B) *E. ocellatus*, C) *D. polylepis* and D) *N. nigricollis*. Antisera were obtained from horses immunized with 1) Montanide, 2) Freund, 3) Carbigen, 4) Emulsigen-D and 5) QH-769. Families of the recognized bands were identified by comparing the electropherograms with those previously published for *B. a. arietans*[23], *E. ocellatus*[24], *D. polylepis*[25] and *N. nigricollis*[26]. Molecular mass markers are depicted to the left.

**Fig. 4:**
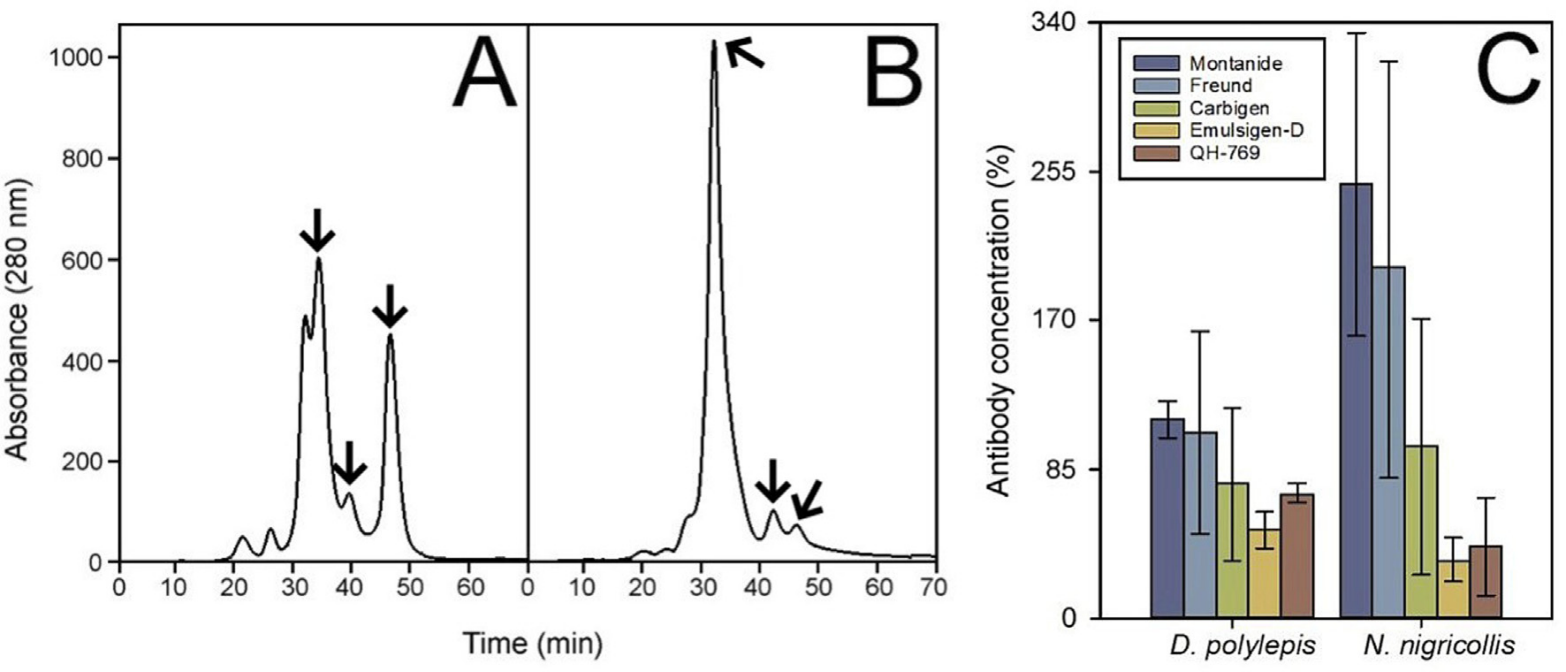
FPLC-chromatograms of venoms of *D. polylepis* (A) and *N. nigricollis* (B), and ELISA determination of the antibody response towards the fractions containing toxins with molecular mass lower that 30 kDa (marked with arrows) at the end of the immunization process (C). ELISA results are expressed as percentage, considering as 100 % the titer of a reference pool of hyperimmune equine sera used for the commercial production of the antivenom EchiTab-plus-ICP. Values correspond to the mean ± standard error (n = 4).

The five adjuvants tested induced an antibody response with a similar reactivity profile towards the four venoms, as judged by Western blot (Fig. 3). However, sera from horses immunized with Montanide or Freund adjuvants were those with the major ability to recognize some low molecular mass components. This was evidenced by a Western blot detecting antibodies towards whole venoms (Fig. 3) and an ELISA detecting antibodies towards the low molecular mass components (<30 kDa) in the venoms of *D. polylepis* and *N. nigricollis* (Fig. 4). This is relevant since PLA_2_s and low molecular mass cytotoxins of the 3FTx family are key components of *N. nigricollis* venom and play a role in the local dermonecrosis characteristic of envenomations by this species of spitting cobra [25], [32].

**Fig. 5:**
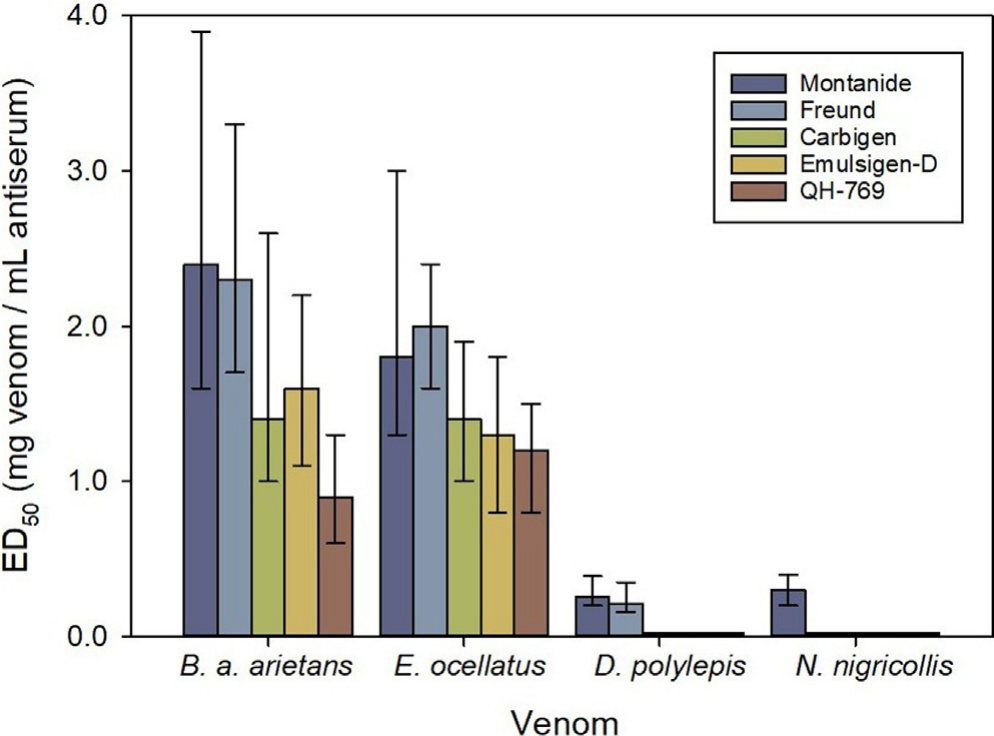
Ability of sera from horses immunized with the four venoms using different adjuvants to neutralize the lethality of the venoms *B. arietans*, *E. ocellatus*, *D. polylepis* and *N. nigricollis*. The sera analyzed correspond to pools obtained at the last point in the immunization scheme. Values correspond to ED_50_ and 95% confidence interval.

In agreement with these immunochemical findings, the pools of sera of horses immunized with Montanide or Freund adjuvants were those with the highest ability to neutralize the lethal activity of all venoms (Fig. 5). However, as indicated by the overlapping of 95 % CIs, this tendency was significant only in some cases such as: Montanide or Freund versus QH-769 in the neutralization of *B. arietans* venom, Freund versus QH-769 in the neutralization of *E. ocellatus* venom, Montanide or Freund versus Carbigen, Emulsigen-D or QH-769 in the neutralization of *D. polylepis* venom, and Montanide versus Freund, Carbigen, Emulsigen-D or QH-769 in the neutralization of *N. nigricollis* venom (Fig. 5). Regardless of the differences between adjuvants, these results agree with the general trend that viperid venoms generate a higher immune response in horses as compared to elapid venoms.

The results obtained in this study agree with previous findings describing that Montanide and Freund are the adjuvants with the highest ability to enhance the antibody response of animals used as immunoglobulin source for antivenom production [6], [7], [8], [9], [10], [33]. However, since these adjuvants induce local damage at the site of injection, their use must be limited to the first stages of the immunization scheme [3]. It remains to be determined whether the low-dose, low-volume, multi-site protocol described for antivenom generation by some authors [10] would reduce the extent of local tissue alterations induced by the emulsion-type adjuvants in horses.

## 4 Conclusions

Our comparative analysis of five emulsion-based adjuvants of different design and composition revealed differences in their ability to generate anti-venom antibody responses in horses as well as in the local tissue damage associated with immunization with venoms of four African snake species. The adjuvants that generated the highest immune response were Montanide and Freund, although they also induced local lesions at the site of injection, with no evidence of systemic alterations. In contrast, less tissue damage was induced by Emulsigen-D and QH-769, although the antibody response in horses receiving these adjuvants was lower. Carbigen induced local lesions similar to Montanide and Freund adjuvants and showed an intermediate ability to enhance the plasma concentration of anti-venom antibodies. Our results suggest that a possible combination of adjuvants aimed at generating a satisfactory immune response without having local deleterious effects could be based on the use of Montanide or Freund-based emulsions in the first immunization steps, followed by the use of Emulsigen-D, QH-769 or similar adjuvants in the following injections, a hypothesis that remains to be investigated.

## Funding

This study was supported by Wellcome Trust [Reference 220517/Z/20/Z] awarded to GL and JMG, and Vicerrectoría de Investigación, Universidad de Costa Rica [projects 741-A0-804 and 741-C0-523].

## Author Contributors

León G and Gutiérrez JM conceptualized and designed the study. Arguedas M, Umaña D, Moscoso E, García A, Pereira C, Sánchez A, Durán G, Cordero D, Sánchez A, Segura Á, Vargas M, Herrera M, Villalta M, Gómez A, Salas C and Díaz C acquired, analyzed and/or interpreted the data. León G drafted the original manuscript. Arguedas M, Umaña D, Moscoso E, García A, Pereira C, Sánchez A, Durán G, Cordero D, Sánchez A, Segura Á, Vargas M, Herrera M, Villalta M, Gómez A, Salas C, Díaz C and Gutiérrez JM reviewed the manuscript and made contributions to the intellectual content. All the authors approved the final version of the manuscript.

## Declaration of Competing Interest

The authors declare that they have no known competing financial interests or personal relationships that could have appeared to influence the work reported in this paper.

## Data Availability

No data was used for the research described in the article.
