# Supplementary material for: Comparison of adjuvant emulsions for their safety and ability to enhance the antibody response in horses immunized with African snake venoms
Source: Vaccine X. 2022 Oct 25;12:100233. doi: 10.1016/j.jvacx.2022.100233 (PMC9634357; doi:10.1016/j.jvacx.2022.100233)
Supplement: Supplementary data 1 [file mmc1.docx]

Supplementary Table S1: Hematological analyses of horses immunized with venoms using different adjuvants

| Adjuvant | Day | HCT^1^ | HGB^2^ | RBC^3^ | WBC^4^ | LYM^5^ | MONO^6^ | GRA^7^ | PLT^8^ |
| --- | --- | --- | --- | --- | --- | --- | --- | --- | --- |
| Montanide | 0 | 45 ± 3 | 15.5 ± 0.9 | 9.3 ± 0.9 | 9.4 ± 0.6 | 3.4 ± 0.6 | 0.7 ± 0.1 | 5.3 ± 1.0 | 166 ± 28 |
|  | 14 | 40 ± 3 | 13.5 ± 1.0 | 8.2 ± 0.9 | 9.5 ± 1.6 | 2.2 ± 0.2 | 0.8 ± 0.3 | 6.5 ± 1.3 | 122 ± 32 |
|  | 28 | 40 ± 3 | 13.5 ± 1.0 | 8.2 ± 0.9 | 12.1 ± 1.0 | 3.6 ± 0.7 | 0.9 ± 0.0 | 7.7 ± 0.8 | 143 ± 68 |
|  | 42 | 38 ± 5 | 12.8 ± 1.7 | 7.8 ± 1.1 | 11.3 ± 0.9 | 3.0 ± 0.5 | 0.8 ± 0.1 | 7.5 ± 1.0 | 146 ± 43 |
|  | 56 | 35 ± 1 | 11.7 ± 0.5 | 7.3 ± 0.3 | 10.1 ± 2.9 | 2.2 ± 0.7 | 0.7 ± 0.1 | 7.3 ± 3.4 | 167 ± 21 |
|  | 70 | 34 ± 2 | 11.4 ± 0.9 | 7.2 ± 0.7 | 12.5 ± 2.2 | 2.9 ± 0.2 | 0.8 ± 0.1 | 8.9 ± 2.2 | 185 ± 33 |
|  | 84 | 35 ± 4 | 11.8 ± 1.2 | 7.4 ± 1.0 | 11.3 ± 2.7 | 2.5 ± 0.8 | 0.7 ± 0.1 | 8.1 ± 3.2 | 207 ± 39 |
| Freund | 0 | 45 ± 4 | 15.4 ± 1.3 | 9.6 ± 0.3 | 10.3 ± 1.9 | 3.0 ± 0.6 | 0.8 ± 0.2 | 6.6 ± 1.5 | 142 ± 85 |
|  | 14 | 43 ± 2 | 14.7 ± 0.4 | 9.1 ± 0.5 | 10.7 ± 0.8 | 2.2 ± 0.9 | 0.8 ± 0.2 | 7.7 ± 0.9 | 113 ± 43 |
|  | 28 | 39 ± 4 | 13.5 ± 1.6 | 8.3 ± 0.5 | 11.6 ± 1.9 | 3.2 ± 0.8 | 0.9 ± 0.2 | 7.5 ± 1.9 | 142 ± 64 |
|  | 42 | 43 ± 4 | 14.6 ± 1.5 | 9.0 ± 0.5 | 12.7 ± 1.0 | 2.6 ± 1.1 | 1.0 ± 0.3 | 9.2 ± 1.8 | 125 ± 77 |
|  | 56 | 39 ± 6 | 13.4 ± 2.1 | 8.4 ± 0.9 | 11.6 ± 2.4 | 2.5 ± 0.6 | 0.9 ± 0.2 | 8.3 ± 2.8 | 140 ± 75 |
|  | 70 | 37 ± 7 | 12.6 ± 2.3 | 8.0 ± 1.1 | 14.1 ± 2.7 | 2.2 ± 0.9 | 0.8 ± 0.2 | 11.1 ± 2.8 | 152 ± 59 |
|  | 84 | 36 ± 4 | 12.4 ± 1.4 | 7.9 ± 0.5 | 12.3 ± 1.1 | 3.2 ± 0.3 | 0.9 ± 0.1 | 8.2 ± 1.2 | 151 ± 60 |
| Carbigen | 0 | 45 ± 1 | 15.5 ± 0.1 | 8.9 ± 0.5 | 10.5 ±1.8 | 3.6 ± 2.2 | 0.8 ± 0.2 | 6.1 ± 1.5 | 136 ± 32 |
|  | 14 | 40 ± 5 | 13.8 ± 1.7 | 7.9 ± 0.8 | 9.9 ± 0.8 | 2.2 ± 1.3 | 0.8 ± 0.2 | 7.0 ± 1.4 | 111 ± 36 |
|  | 28 | 40 ± 2 | 13.8 ± 0.7 | 8.1 ± 0.9 | 11.5 ± 1.7 | 3.1 ± 1.8 | 0.9 ± 0.3 | 7.5 ± 1.7 | 151 ± 27 |
|  | 42 | 44 ± 3 | 15.0 ± 0.8 | 8.9 ± 0.9 | 11.7 ± 2.4 | 3.4 ± 2.2 | 0.8 ± 0.2 | 7.5 ± 0.7 | 140 ± 25 |
|  | 56 | 39 ± 2 | 13.5 ± 0.7 | 8.1 ± 0.7 | 10.8 ± 2.9 | 3.2 ± 1.6 | 0.8 ± 0.3 | 6.9 ± 2.1 | 131 ± 19 |
|  | 70 | 41 ± 3 | 13.9 ± 1.1 | 8.4 ± 1.0 | 12.5 ± 2.3 | 3.8 ± 1.6 | 1.0 ± 0.3 | 7.8 ± 0.9 | 164 ± 21 |
|  | 84 | 41 ± 4 | 13.8 ± 0.9 | 8.3 ± 0.8 | 12.0 ± 2.3 | 3.7 ± 1.6 | 0.9 ± 0.3 | 7.4 ± 1.0 | 176 ± 22 |
| Emulsigen  D | 0 | 46 ± 4 | 16.1 ± 1.4 | 9.4 ± 0.8 | 9.1 ± 0.9 | 2.7 ± 0.8 | 0.6 ± 0.1 | 5.8 ± 0.4 | 134 ± 32 |
|  | 14 | 48 ± 1 | 16.3 ± 0.3 | 9.7 ± 0.3 | 10.6 ± 0.3 | 2.1 ± 0.9 | 0.8 ± 0.3 | 7.7 ± 1.4 | 110 ± 34 |
|  | 28 | 42 ± 2 | 14.4 ± 0.4 | 8.5 ± 0.2 | 11.6 ± 1.1 | 2.9 ± 1.2 | 0.9 ± 0.3 | 7.8 ± 0.6 | 131 ± 74 |
|  | 42 | 45 ± 1 | 15.5 ± 0.5 | 9.2 ± 0.3 | 10.3 ± 1.5 | 2.7 ± 1.2 | 0.8 ± 0.2 | 6.9 ± 0.5 | 131 ± 64 |
|  | 56 | 43 ± 3 | 14.5 ± 0.8 | 8.6 ± 0.3 | 10.1 ± 1.2 | 2.4 ± 1.3 | 0.7 ± 0.1 | 7.0 ± 0.8 | 107 ± 69 |
|  | 70 | 40 ± 1 | 13.6 ± 0.6 | 8.1 ± 0.4 | 10.5 ± 1.1 | 2.9 ± 0.8 | 0.7 ± 0.2 | 6.9 ± 0.4 | 151 ± 43 |
|  | 84 | 42 ± 3 | 14.2 ± 0.6 | 8.5 ± 0.3 | 8.8 ± 0.6 | 2.4 ± 0.4 | 0.6 ± 0.2 | 5.9 ± 0.1 | 156 ± 29 |
| QH769 | 0 | 44 ± 4 | 15.0 ± 1.4 | 8.7 ± 0.9 | 9.3 ± 0.8 | 3.6 ± 0.3 | 0.8 ± 0.1 | 5.0 ± 0.7 | 132 ± 14 |
|  | 14 | 43 ± 2 | 14.6 ± 0.7 | 8.4 ± 0.6 | 9.5 ± 0.9 | 3.1 ± 0.5 | 0.8 ± 0.2 | 5.7 ± 0.7 | 82 ± 13 |
|  | 28 | 41 ± 3 | 14.1 ± 1.0 | 8.1 ± 0.5 | 11.1 ± 2.2 | 3.5 ± 0.7 | 0.7 ± 0.1 | 6.9 ± 2.6 | 128 ± 7 |
|  | 42 | 43 ± 3 | 14.7 ± 0.8 | 8.5 ± 0.6 | 10.1 ± 1.5 | 3.4 ± 0.1 | 0.8 ± 0.1 | 5.9 ± 1.5 | 123 ± 23 |
|  | 56 | 40 ± 4 | 13.6 ± 1.2 | 7.8 ± 0.8 | 9.2 ± 1.2 | 3.6 ± 0.3 | 0.8 ± 0.1 | 4.8 ± 1.2 | 126 ± 22 |
|  | 70 | 40 ± 6 | 13.3 ± 1.8 | 7.8 ± 1.1 | 9.8 ± 1.4 | 3.6 ± 0.5 | 0.9 ± 0.1 | 5.3 ± 1.1 | 129 ± 8 |
|  | 84 | 39 ± 3 | 13.4 ± 1.2 | 7.8 ± 0.8 | 9.1 ± 1.3 | 3.2 ± 0.3 | 0.8 ± 0.1 | 5.2 ± 1.1 | 118 ± 14 |

^1^ HCT: Hematocrit. Values are expressed as percentage, and correspond to the average ± SD.

^2^ HGB: Hemoglobin. Values are expressed as g/dL, and correspond to the average ± SD.

^3^ RBC: Erythrocyte. Values are expressed as cells x 10^6^/μL, and correspond to the average ± SD.

^4^ WBC: Leukocyte. Values are expressed as cells x 10^3^/μL, and correspond to the average ± SD.

^5^ LYM: Lymphocyte. Values are expressed as cells x 10^3^/μL, and correspond to the average ± SD.

^6^ MONO: Monocyte. Values are expressed as cells x 10^3^/μL, and correspond to the average ± SD.

^7^ GRA: Granulocyte. Values are expressed as cells x 10^3^/μL, and correspond to the average ± SD.

^8^ PLT: Platelet. Values are expressed as cells x 10^3^/μL, and correspond to the average ± SD.
